# Social Determinants of Health and Health Equity in the Diagnosis and Management of Pediatric Mild Traumatic Brain Injury: A Content Analysis of Research Underlying Clinical Guidelines

**DOI:** 10.1089/neu.2023.0021

**Published:** 2023-09-29

**Authors:** Nathan E. Cook, Alicia Kissinger-Knox, Ila A. Iverson, Brian C. Liu, Charles E. Gaudet, Marc A. Norman, Grant L. Iverson

**Affiliations:** ^1^ Department of Physical Medicine and Rehabilitation, Harvard Medical School, Boston, Massachusetts, USA.; ^2^ MassGeneral Hospital for Children Sports Concussion Program, Waltham, Massachusetts, USA.; ^3^ Department of Physical Medicine and Rehabilitation, Spaulding Rehabilitation Hospital, Charlestown, Massachusetts, USA.; ^4^ Department of Psychology, University of British Columbia, Vancouver, British Columbia, Canada.; ^5^ Department of Psychiatry, UC San Diego School of Medicine, San Diego, California, USA.; ^6^ Department of Physical Medicine and Rehabilitation, Shoen Adams Research Institute at Spaulding Rehabilitation, Charlestown, Massachusetts, USA.

**Keywords:** brain injury, concussion, pediatric, socioeconomic status

## Abstract

We conducted a content analysis of the literature underlying the Centers for Disease Control and Prevention (CDC) Guideline on the Diagnosis and Management of Mild Traumatic Brain Injury Among Children (i.e., the “Guideline”) to determine the extent to which social determinants of health (SDoH) were examined or addressed. The systematic review forming the basis for the Guideline included 37 studies addressing diagnosis, prognosis, and treatment/rehabilitation. We examined those studies to identify SDoH domains derived from the U.S. Department of Health and Human Services' Healthy People 2020 and 2030 websites. No study explicitly mentioned “social determinants of health,” by name, and few studies addressed SDoH domains as a primary focus (ranging from 0% to 27% of studies across SDoH domains). The most frequently represented SDoH domains, described in an inferential or a descriptive manner, were Education Access and Quality (29.7% of studies), Social and Community Context (27.0% of studies), and Economic Stability (21.6% of studies). Health Care Access (13.5% of studies) was less well represented and no studies (0%) examined Neighborhood and Built Environment. In terms of the CDC clinical questions, SDoH were only examined as predictors of outcome (prognosis) and no studies examined SDoH in relation to diagnosis or treatment/rehabilitation. The Guideline includes some commentary on health literacy and socioeconomic status. Overall, social determinants of health are largely unrepresented as important or meaningful variables influencing the Guideline on the Diagnosis and Management of Mild Traumatic Brain Injury Among Children, or in the studies that informed the Guideline.

## Introduction

Social and economic circumstances significantly influence children's health and well-being.^1,2^ Social determinants of health (SDoH) refer to the environments and conditions in which we are born, raised, educated, live, and work that influence individual and group differences in health status and outcomes.^3^ Several SDoH domains have been identified including, Economic Stability (e.g., poverty, ability to afford healthcare, and housing instability), Education Access and Quality (e.g., language and literacy, high school graduation, and enrollment in higher education), Health Care Access and Quality (e.g., access to healthcare including primary care, health literacy, and health and dental insurance), Neighborhood and Built Environment (e.g., environmental conditions such as safe air and water, biking and walking accessibility, and crime and violence), and Social and Community Context (e.g., experiences of discrimination or racism, parental mental health, and positive versus negative relationships within the family and community).^4,5^ Social determinants are causal factors in health inequity.

Health equity means that all people should have fair and equal opportunity to enjoy their full health potential^6^ and health inequities are systematic differences in health status between groups, with significant social and economic costs both to individuals and societies.^7^ Sociocultural and demographic factors associated with health inequity (that partially overlap with and are associated with SDoH in direct and indirect ways) include race, ethnicity, language, culture, and socioeconomic status (SES). For example, disparities in pediatric healthcare access and health outcomes have been reported in association with race and ethnicity,^8-10^ SES,^11^ English language proficiency,^12^ and acculturation.^13,14^

There are important associations between SDoH, health inequity, and pediatric injury. For example, social gradients have been found with regard to causes of medically treated adolescent injuries, such that higher income is associated with greater likelihood of sport-related injury whereas poverty is associated with greater likelihood of injuries due to fighting.^15^ Moreover, children from low-income households, children from families that receive income assistance, and children born to teenage mothers are at an elevated risk for burn injuries.^16^

A pediatric injury that is a major source of concern for parents, healthcare providers, school personnel, and legislators is concussion, or mild traumatic brain injury (mTBI).^17–23^ Approximately 1 in 100 school-aged children in the United States sustain a mTBI each year.^24^ Emerging research findings have begun to document the clear and expected relevance of SDoH with regard to pediatric mTBI. For example, there is accumulating evidence of racial, ethnic, and socioeconomic disparities in accessing concussion care,^25-27^ recovery time, and supports received upon returning to school following the injury,^28,29^ as well as health literacy (i.e., general concussion knowledge, familiarity with concussion laws, and awareness of concussion symptoms).^30-32^

Given the significance of concussion and mTBI as a public health concern, the Centers for Disease Control and Prevention (CDC) undertook a massive and pioneering effort to develop the first clinical management guideline in the United States for pediatric mTBI, which was published as the Centers for Disease Control and Prevention Guideline on the Diagnosis and Management of Mild Traumatic Brain Injury Among Children (i.e., the “Guideline”).^33^ The Guideline includes 19 sets of recommendations regarding the diagnosis, prognosis, and management/treatment of pediatric mTBI. The recommendations in the Guideline were developed based on clinical science identified in a major systematic review.^34^

The systematic review was organized around six clinical questions related to: 1) diagnosing mTBI; 2) accuracy of routine head imaging; 3) features associated with increased risk of intracranial injury/abnormal head imaging; 4) factors associated with worse outcome in the first year following mTBI; 5) factors associated with worse outcome beyond 1 year following mTBI; and 6) treatments associated with improved mTBI outcome. The systematic review screened 15,150 articles, a total of 2984 full-text articles were reviewed, and 75 articles were ultimately included in the synthesis across the clinical questions (some articles were reviewed across multiple clinical questions). The CDC established a multi-disciplinary workgroup that drafted recommendations relevant to each of the six clinical questions based on the studies identified in the systematic review, along with related evidence, scientific principles, and expert inference.^33^ Authors from multiple disciplines including primary care pediatrics, athletic training, physical therapy, and sports medicine have highlighted the broad applicability and importance of the Guideline.^35–38^

The Guideline does not reference “social determinants” or “health equity” by name. However, the Guideline mentions and discusses aspects of SDoH. For instance, the Guideline notes the importance of health literacy and social support as means of promoting better patient outcomes. In addition, family and social stressors (Recommendation 8B), race, ethnicity, and SES (Recommendation 9B) are highlighted as potentially associated with worse outcome following injury and thus healthcare professionals are encouraged to monitor youth with these and other risk factors more closely (Recommendation 11A).

The prevailing Guideline for the diagnosis and management of mTBI in children represents an extraordinary synthesis of a broad and diverse literature leading to specific and practical recommendations for healthcare providers. Leveraging that multi-year effort from multi-disciplinary experts, the purpose of this paper was to carefully review the studies identified in the systematic review^34^ and relied upon to support the practice recommendations in the Guideline,^33^ and to extract information pertaining to SDoH and health equity. More specifically, we conducted a content analysis to determine the extent to which the clinical science underlying the Guideline for diagnosing and managing pediatric mTBI has examined or discussed SDoH or health equity. Further, we sought to identify critical knowledge gaps to encourage and suggest future directions to enhance the incorporation of SDoH in pediatric mTBI research and clinical practice.

## Methods

### Selection of studies

We selected studies underlying the CDC Guideline,^33^ which were identified through a systematic review.^34^ We collected the articles identified for four of the six clinical questions from the systematic review (Questions 1, 4, 5, and 6 on the topics of diagnosis, short-term prognosis [within 1 year of injury], longer-term prognosis [beyond 1 year post-injury], and treatment/rehabilitation, respectively). Our focus was on diagnosis and management; thus, we did not examine the studies relevant to the two clinical questions that addressed neuroimaging. A total of 38 studies were identified; one study was excluded because it was a conference presentation, resulting in a final sample of 37 published studies.^39–75^

### Data extraction

We developed a coding sheet to identify SDoH and associated subcategories derived from both the Healthy People 2020^5^ and Healthy People 2030^4^ websites. Each of these websites lists the same five SDoH domains: Economic Stability, Education Assess and Quality, Health Care Access and Quality, Neighborhood and Built Environment, and Social and Community Context. Healthy People 2020 provided a bulleted list of these five domains, with several key issues and underlying factors within each domain. For example, a key issue listed under Economic Stability is “poverty.” We included each of the key issues and underlying factors as separate variables to code. We then cross-referenced this list with the information on Healthy People 2030, which provides a separate webpage for each SDoH domain, with an “Overview and Objectives” section that presents brief text descriptions. Three authors (NEC, AKK, BCL) read each text description and extracted any additional key issues or underlying factors that were not included in the Healthy People 2020 bulleted list. For example, in the Health Care Access and Quality domain, health and/or dental insurance was listed in Healthy People 2030, but it was not on the Healthy People 2020 list; therefore, it was added to the coding sheet.

The coding sheet also included summary information about the study sample including the sample size, average age, age range, as well as the gender, racial, and ethnic composition of the sample (coders collected the exact sample details or identified that the information was “Not Reported” by the study authors). The coding sheet also included study design, whether studies included exclusionary criteria based on demographic, sociocultural, or health factors, and whether studies mentioned future directions/research needs regarding social determinants or health equity. Lastly, we added summary variables to characterize whether, or the extent to which, each study analyzed or provided information about the following five key health equity variables: race, ethnicity, culture/acculturation, SES, and language. Coders could indicate whether the study: 1) provided no mention of the variable; 2) included the variable as a demographic category only; or 3) examined the variable in depth (e.g., the health equity variable was a primary variable of interest in the study or outcome/prognostic results were stratified and reported across levels of the variable). The study coding sheet is included in the online supplemental materials.

After the coding sheet was developed, four authors (NEC, AKK, BCL, IAI) reviewed the 37 articles. Five studies were assigned to all four raters as training articles and the raters met as a group to discuss and calibrate ratings. The remaining 32 articles were assigned to two raters each. Authors performed a content analysis independently by extracting details for each study and completing the coding sheet described above. Discrepancies were resolved by discussion. Lastly, two authors (NEC and AKK) independently determined whether articles addressed SDoH by examining the variables in an inferential or intentional way (e.g., the SDoH variable represented a primary focus or emphasis of the study, or the SDoH variable served as a primary predictor of outcome), or in a descriptive or demographic way (e.g., the SDoH variable was summarized as a demographic variable only, mentioned in the discussion section as an area of future study, or utilized as a design feature of the study such as might pertain to recruitment, inclusion/exclusion criteria).

### Data analysis and synthesis

The percentages of studies that were identified as including each of the five SDoH domains and each of the five key health equity variables were calculated and summarized descriptively. No statistical significance testing or quantitative synthesis/meta-analytic techniques were used.

## Results

The 37 studies included a total of 15,887 participants (median = 150 participants; range 30-3091). The mean sample age was reported in 14 studies and the average sample age was 12.9 years (standard deviation = 3.2). Samples included individuals ranging in age from 0 to 22 years, although the median age for the lower bound of age ranges was 6 years and the median upper bound was 16 years. Gender was reported in nearly all studies (k = 34; 91.9%). Racial composition of the samples was only reported in about one-third of studies (k = 11; 29.7%). Seven of these 11 studies and one additional study that did not report racial composition reported ethnic composition of the samples (k = 8; 21.6%). Study samples were mostly recruited from hospitals/emergency departments (k = 30; 81.1%), with very few studies recruiting from high school sports (k = 3; 8.1%) or specialty concussion clinics (k = 3; 8.1%). One study (2.7%) reported recruiting broadly from a healthcare system that included the emergency department, hospital admissions, and outpatient clinics. No study explicitly recruited from pediatric primary care. Study designs included prospective cohort (k = 26; 70.3%), retrospective cohort (k = 4; 10.8%), retrospective case-control (k = 3; 8.1%), and randomized controlled trials (k = 2; 5.4%), with one cross-sectional cohort (2.7%) and one cross-sectional case–control (2.7%).

### Social determinants of health

**FIG. 1.**
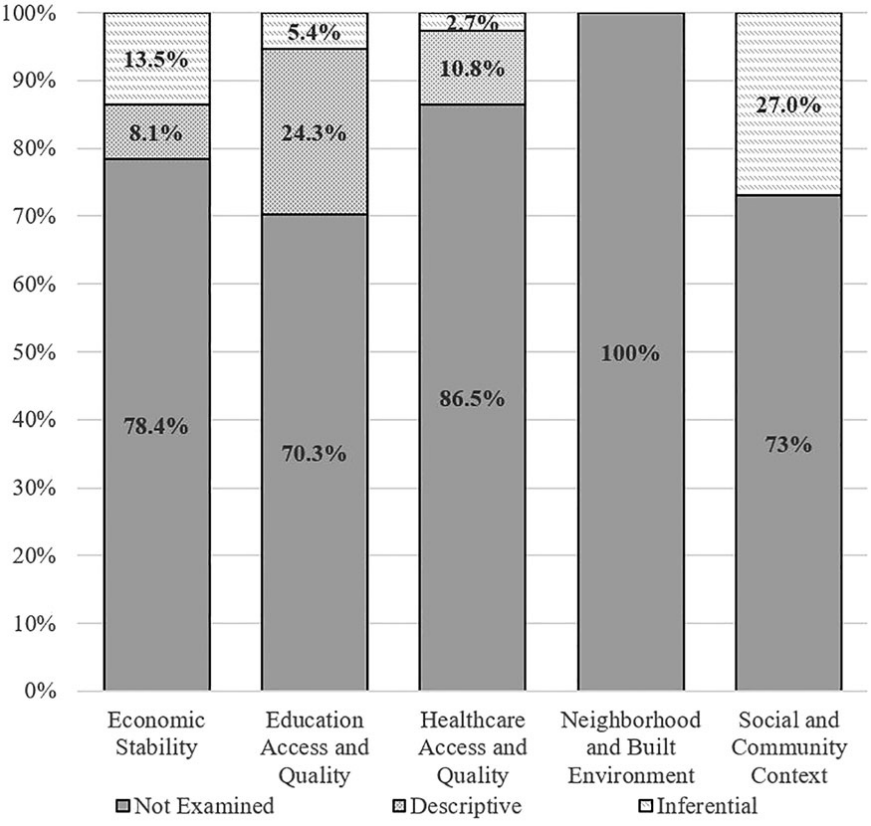
Proportion of studies examining social determinants of health domains.

No study explicitly mentioned “social determinants of health,” by name. The content analysis results related to SDoH domains among the 37 included studies are summarized in Table 1 and Figure 1. The most common SDoH domains addressed in an inferential or intentional manner were Social and Community Context (27.0% of studies), followed by Economic Stability (13.5%). Very few studies addressed Education Access and Quality (5.4%) or Healthcare Access and Quality in an inferential (2.7%) fashion. No studies examined the SDoH domain Neighborhood and Built Environment, either in an inferential or a descriptive manner. Overall, about two-thirds of the studies (67.5%) either did not address a SDoH domain at all or addressed a SDoH domain in a descriptive or demographic fashion (e.g., as a demographic variable only, mentioned in the discussion section as an area of future study, as a design feature of the study such as might pertain to recruitment, inclusion/exclusion criteria). In terms of the CDC clinical questions, SDoH were only examined as predictors of outcome (prognosis) and no studies examined SDoH in relation to diagnosis or treatment/rehabilitation.

**Table 1.** Summary of Content Analysis Results Regarding Inclusion of Social Determinants of Health

First author (year)	CDC question(s)		Social determinants of health (SDoH)				
			Economic stabilty	Education access and quality	Health care access and quality	Neighborhood and built environment	Social and community context
Agrawal (2005)	4	Outcome <1 year	–	Descriptive	–	–	–
Babikian (2011)	4	Outcome <1 year	–	Descriptive	Descriptive	–	–
Babikian (2013)	5	Outcome ≥1 year	–	Inferential	–	–	Inferential
Barlow (2010)	4,5	Outcome <1 year and ≥1 year	–	–	–	–	Inferential
Berger (2002)	1	Diagnosis	–	–	–	–	–
Blume (2012)	4	Outcome <1 year	Descriptive	–	Descriptive	–	–
Bouvier (2012)	4	Outcome <1 year	–	–	–	–	–
Castile (2012)	4	Outcome <1 year	–	–	–	–	–
Chrisman (2013)	4	Outcome <1 year	–	–	—	–	–
Fay (2010)	5	Outcome ≥1 year	–	Descriptive	–	–	–
Gagnon (2004)	1	Diagnosis	–	–	Descriptive	–	–
Geyer (2009)	1	Diagnosis	–	–	–	–	–
Grubenhoff (2010)	1	Diagnosis	–	Descriptive	–	–	–
Hessen (2008)	5	Outcome ≥1 year	Descriptive	Descriptive	–	–	–
Levin (2008)	4,5	Outcome <1 year and ≥1 year	Inferential	–	–	–	–
Lovell (2003)	1	Diagnosis	–	–	–	–	–
Lumba-Brown (2014)	6	Treatment	–	–	–	–	–
Massagli (2004)	5	Outcome ≥1 year	–	–	–	–	–
Max (2013b)	4	Outcome <1 year	Inferential	–	–	–	Inferential
Max (2013a)	5	Outcome ≥1 year	Inferential	–	–	–	Inferential
Moran (2009)	4	Outcome <1 year	–	–	–	–	–
Mucha (2014)	1	Diagnosis	–	–	–	–	–
O'Connor (2012)	4,5	Outcome <1 year and ≥1 year	–	Inferential	–	–	Inferential
Olsson (2013)	4,5	Outcome <1 year and ≥1 year	–	–	–	–	Inferential
Papoustis (2014)	5	Outcome ≥1 year	–	–	–	–	–
Ponsford (1999)	4	Outcome <1 year	–	Descriptive	–	–	Inferential
Ponsford (2001)	6	Treatment	–	Descriptive	–	–	Inferential
Rivara (2011)	4,5	Outcome <1 year and ≥1 year	Inferential	–	Descriptive	–	Inferential
Schatz (2006)	1	Diagnosis	–	Descriptive	–	–	–
Smyth (2014)	4,5	Outcome <1 year and ≥1 year	–	–	–	–	Inferential
Taylor (2015)	5	Outcome ≥1 year	Descriptive	Descriptive	–	–	–
Teasdale (2003)	5	Outcome ≥1 year	–	–	–	–	–
Thomas (2015)	6	Treatment	–	–	–	–	–
van der Veek (2015)	4	Outcome <1 year	–	–	–	–	–
Yeates (1999)	4	Outcome <1 year	–	–	–	–	–
Zonfrillo (2014)	4,5	Outcome <1 year and ≥1 year	Inferential	–	Inferential	–	–
Zuckerman (2012)	4	Outcome <1 year	–	–	–	–	–

– = Not addressed.

Inferential = SDoH variable represented in an inferential or intentional way (e.g., a primary focus or emphasis of the study, a primary predictor of outcome).

Descriptive = SDoH variable represented in a descriptive or demographic way (e.g., summarized as a demographic variable only, mentioned in the discussion section as an area of future study, or utilized as a design feature of the study such as might pertain to recruitment, inclusion/exclusion criteria, etc.).

CDC, Centers for Disease Control and Prevention.

### Health equity factors

**FIG. 2.**
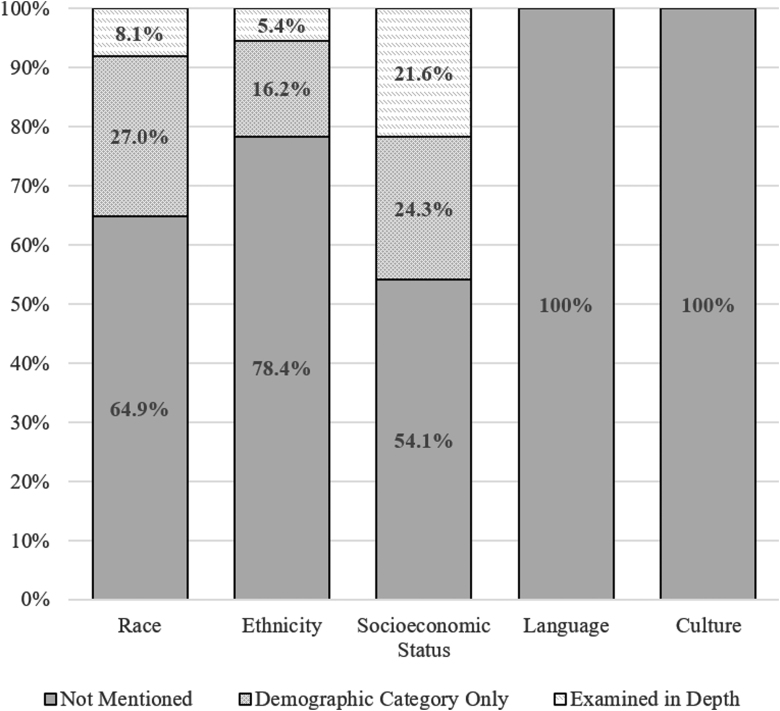
Proportion of studies examining health equity variables.

No study explicitly mentioned “health equity” or similar phrasing. The term “disparities” appeared in three studies, but only one study used the term in relation to health disparities or health equity (one study described “disparities” with regard to differing results from adjusted versus unadjusted statistical analyses and another study noted “gender disparities” with regard to biomechanics in head and neck acceleration). The content analysis results related to health equity factors among the 37 included studies are summarized in Table 2 and Figure 2. Approximately one in five studies (k = 8; 21.6%) examined a health equity variable in depth, such as by investigating SES as a direct predictor or modifier of outcome. Another third of the studies (32.4%) mentioned at least one of the health equity factors, but only as a demographic category (e.g., reporting the racial or ethnic composition of the sample). The most commonly examined health equity factor was SES, with roughly half the studies (45.9%) including SES as either a demographic category or examining it in depth. No studies examined or reported on culture/acculturation or language.

**Table 2.** Summary of Content Analysis Results Regarding Health Equity Factors, Exclusionary Criteria, and Future Directions

First author (year)	Health equity factors					Exclusion criteria^*^	Future direcitons^^^
	Race	Ethnicity	SES	Culture	Language		
Agrawal (2005)	–	–	–	–	–	Yes	No
Babikian (2011)	Demographic	Demographic	Demographic	–	–	Yes	No
Babikian (2013)	–	–	Demographic	–	–	No	No
Barlow (2010)	–	–	Demographic	–	–	No	No
Berger (2002)	Demographic	–	–	–	–	No	No
Blume (2012)	Demographic	Demographic	Demographic	–	–	No	No
Bouvier (2012)	–	–	–	–	–	No	No
Castile (2012)	–	–	–	–	–	No	No
Chrisman (2013)	–	–	–	–	–	No	No
Fay (2010)	In depth	–	In depth	–	–	Yes	Yes
Gagnon (2004)	–	–	–	–	–	Yes	No
Geyer (2009)	–	–	–	–	–	No	No
Grubenhoff (2010)	–	–	–	–	–	Yes	No
Hessen (2008)	–	–	–	–	–	No	No
Levin (2008)	–	–	In depth	–	–	Yes	No
Lovell (2003)	–	–	–	–	–	Yes	No
Lumba-Brown (2014)	–	–	–	–	–	Yes	No
Massagli (2004)	Demographic	–	–	–	–	Yes	No
Max (2013)	Demographic	Demographic	Demographic	–	–	Yes	Yes
Max (2013)	In depth	In depth	In depth	–	–	Yes	No
Moran (2009)	Demographic	–	Demographic	–	–	Yes	No
Mucha (2014)	–	–	–	–	–	Yes	No
O'Connor (2012)	Demographic	Demographic	In depth	–	–	No	No
Olsson (2013)	–	–	–	–	–	Yes	No
Papoustis (2014)	–	–	In depth	–	–	Yes	No
Ponsford (1999)	–	–	Demographic	–	–	Yes	No
Ponsford (2001)	–	–	Demographic	–	–	Yes	Yes
Rivara (2011)	Demographic	Demographic	In depth	–	–	No	No
Schatz (2006)	–	–	–	–	–	No	No
Smyth (2014)	–	Demographic	–	–	–	No	Yes
Taylor (2015)	Demographic	–	In depth	–	–	Yes	No
Teasdale (2003)	–	–	–	–	–	No	No
Thomas (2015)	–	–	–	–	–	Yes	No
van der Veek (2015)	–	–	–	–	–	No	No
Yeates (1999)	Demographic	–	Demographic	–	–	Yes	Yes
Zonfrillo (2014)	In depth	In depth	In depth	–	–	No	Yes
Zuckerman (2012)	–	–	–	–	–	Yes	No

^*^ Studies included exclusionary criteria based on demographic, sociocultural, or health factors.

^Studies mentioned future directions/research needs regarding social determinants or health equity.

SES, socioeconomic status.

### Demographic, sociocultural, or health factors as exclusionary criteria

Over half of the studies (k = 20; 54.1%) excluded participants based on demographic, sociocultural, or health factors, namely language proficiency and the presence of pre-injury health conditions/disabilities. Many studies excluded children with limited English proficiency,^51,62–65,69,71,75^ or if a guardian/caregiver was unable to complete the research consent process in English.^65,69,71^ One study included English speaking children of Spanish speaking parents.^51^ Many studies excluded children with pre-injury health conditions or disabilities, such as a history of a developmental delay, disability, or disorder,^56,63,76,77^ including autism spectrum disorder,^57,58,75,78^ intellectual disability,^57-59,69,71,73,79^ learning disabilities, including attention-deficit/hyperactivity disorder (ADHD),^49,54,71,75^ enrollment in special education,^49,75^ or diagnosed psychiatric or psychological disorder^60^ including schizophrenia,^57,58,78^ severe psychiatric history including hospitalization,^59,69,73,75,79^ or behavioral problems.^49^ No studies provided rationale or explanation for such exclusionary criteria, nor described such exclusions as a limitation of their study, such that the generalizability of the reported results might be limited. Only one study emphasized the importance of assessing sociocultural factors, such as a youth's pre-injury family background.^41^

### Social determinants or health equity as future clinical and research considerations

No study explicitly mentioned social determinants of health, by name, as an important framework to guide future mTBI research. One study (2.7%) referenced the need for future research to further explore health disparities in relation to outcome following brain injury, such as disparities in care, adherence to treatment and rehabilitation plans, and limitations in social support among disadvantaged children and families, though the authors specifically mention disparities in outcome following severe TBI, as opposed to mTBI.^74^ These authors further implore healthcare providers to “ensure multidimensional support and resources” and attend to such factors as socioeconomic disadvantage when providing clinical care for youth with TBI, specifically referencing those who are at socioeconomic disadvantage, including families with lower parental education, lower SES, and Medicaid insurance.^74^ A few other studies (k = 5; 13.5%) mentioned or referred to SDoH or health equity indirectly. For example, one study recommended that future research examine family functioning and parental adjustment in relation to outcome and recovery following pediatric mTBI.^73^ Another study referred generally to “preinjury life stressors” and “a history of previous stressful life events” as important to consider in both clinical and research considerations regarding pediatric mTBI.^68^ One study implied the importance of health literacy by recommending that information and suggested coping strategies be provided to children and their parents following pediatric mTBI.^65^ Three studies highlighted factors such as disability status (i.e., neurodevelopmental disorders)^73,79^ and SES^57^ as important variables to address in terms of sample inclusion and matching criteria.

## Discussion

This content analysis found that SDoH and health equity have been significantly underrepresented in the clinical research literature underlying the CDC Guideline on the Diagnosis and Management of Mild Traumatic Brain Injury Among Children. Studies underlying the Guideline predominantly addressed only a subset of SDoH domains, most commonly Social and Community Context (mainly examining positive vs. negative relationships at home, four studies examining parental mental health, and two studies examining parental incarceration) and Education Access and Quality (e.g., parental education level, child's pre-injury learning disability status, and child's school functioning). The remaining SDoH domains are of importance to pediatric mTBI management but have very little representation in the literature.

For example, Economic Stability is a major domain of interest given emerging evidence of disparities in accessing concussion care.^25-27^ Health Care Access and Quality is also an important area for future research given initial evidence of health literacy disparities relating to general concussion knowledge, familiarity with concussion laws, and awareness of concussion symptoms.^30-32^ Moreover, no studies examined Neighborhood and Built Environment, which is of significant interest when considering the scientific support for exercise-based concussion rehabilitation, which might include, for some youth, the ability to safely access outdoor spaces to be physically active. Regarding the clinical questions that form the basis for the Guideline, no studies addressing diagnosis (clinical question 1) and only one study addressing treatment (clinical question 6) included SDoH in an inferential or primary fashion. It is important to note that our approach to coding SDoH was liberal and might overrepresent the extent to which social determinants have been examined or woven into this literature.

This content analysis also examined the extent to which health equity and related sociocultural variables were represented or discussed. Health equity was not mentioned explicitly by name, and it was not represented as a primary framework by which researchers designed studies or interpreted results in nearly all of the studies included in the Guideline. Only one study described its findings as revealing disparities in outcomes among disadvantaged youth and encouraged future work to investigate mTBI-related health disparities. Nearly half of the studies did not mention race, ethnicity, language, or culture/acculturation in any context, not even as a demographic characteristic describing the sample composition. Only about one in five studies examined a health equity variable in depth and most frequently this involved SES, such as by including SES as a predictor of outcome. Race and ethnicity were rarely examined in depth (only three studies examined either race and/or ethnicity). No studies underlying the pediatric mTBI Guidelines examined or represented culture/acculturation or language as contributors to health inequity.

Just over half the studies underlying the prevailing Guideline utilized exclusionary criteria based on demographic, sociocultural, or health factors. Specifically, studies excluded youth from participating based on English language proficiency and/or the presence of pre-injury health conditions/disabilities. Moreover, no study discussed these exclusionary criteria as a limitation nor provided a rationale or explanation for the exclusion. Language-based exclusionary criteria may reflect resource limitations on researchers' part, such as limited funding to adequately translate informed consent documents in multiple languages or the lack of availability of interpreters to ensure appropriate informed consent procedures. Regardless of potential rationales or practical considerations from a research perspective, these exclusionary criteria represent clear limitations to the generalizability of findings, such that youth from underprivileged backgrounds are under-represented in the literature and the extent to which some of the findings from the literature apply to them is unknown.

Lastly, no study explicitly mentioned SDoH, health equity, or similar phrasing as an important framework to guide future mTBI research. Only one study explicitly mentioned the need for future health disparities research regarding clinical care and outcomes, but the authors were referencing severe TBI, not mTBI. A few SDoH and health equity factors were mentioned indirectly, such as authors noting the need for future research to explore family functioning and adjustment following pediatric mTBI, or pre-injury stressful life events in relation to outcome from mTBI.

It is important to note that the articles included in this content analysis, and that form the basis for the Guideline, were drawn from a systematic review of literature published between 1990 and mid-2015.^34^ It is hoped that more recent literature will increasingly incorporate and examine SDoH and health equity. In turn, the literature base that informs future updated guidelines will more adequately address SDoH and health equity. Moreover, the systematic review that formed the basis for the Guideline did not include a specific question regarding SDoH or health equity, and thus the representation of SDoH and health equity in the larger pediatric mTBI literature may differ, to some degree, from the studies included in this content analysis. Our focus, however, was to review specifically the articles that formed the basis for the development of the current Guideline.

### Clinical implications

Clinicians treating children with concussion are encouraged to carefully consider how SDoH might be relevant or important during clinical assessment, patient/family education, and during treatment or rehabilitation, and incorporate relevant information into their care, treatment, and rehabilitation plans. For example, for a youth with persistent symptoms following concussion, clinical science strongly supports the use of exercise-based active rehabilitation.^80-82^ However, social considerations such as SES and resource availability are crucial to consider. For many youth, access to an in-home treadmill or exercise bike is not feasible or realistic for economic, space, or other reasons. Additionally, some youth may lack access to fitness facilities with such equipment. Moreover, even suggestions for youth to exercise outdoors may require modification, such as those who live in neighborhoods with limited green space or parks, on or near busy roadways that would not be safe and conducive to jogging, or in neighborhoods where being outside is unsafe in terms of potential for violence.

The reading level and readability scores of patient education materials is an important consideration. Parental literacy and educational attainment may reduce a family's ability to follow recommendations offered by healthcare providers. It is also important to have access to medical interpreters and to have educational materials and supports available in primary languages for the patients served.

For youth experiencing prolonged recovery following mTBI, referral to specialty care is advised.^83^ However, facilitating referrals to specialty clinics is often a complex process and families may be poorly equipped to navigate the complexities of identifying specialty clinics, seeking referrals, and dealing with health insurance policies. Recent research indicates that specialty concussion clinic patients are more likely to: 1) have private insurance and 2) be White.^84^ Recommendations or referrals for additional forms of treatments to support concussion recovery such as physical therapy or mental health also may present challenges for families with economic constraints or insurance-based limitations. Clinicians are encouraged to proactively support families and facilitate their engagement in care by making direct contact with referral providers to the greatest extent possible.

Youth often require time-limited school accommodations during concussion recovery. However, pursuing and securing accommodations by communicating with school personnel might be more or less challenging for certain caregivers based on factors such as comfort with the school support personnel structure, knowledge of how to contact school nurses or guidance counselors (and comfort in doing so), or trust in the school team that the needs of their children will be prioritized and met.

### Implications for future research

There is a need to emphasize SDoH and health equity in pediatric mTBI research and to refine our understanding of how such factors relate to diagnosing, treating, and managing this injury. From a methodological perspective, concussion researchers should make concerted efforts to use qualitative and community-based participatory research designs and methodologies. These methodologies have proven particularly beneficial in terms of elucidating health disparities as well as conceptualizing and designing ways to intervene upon social determinants to reduce disparities among vulnerable groups.^85,86^ Further, research teams are encouraged to partner with school-based athletic trainers, healthcare providers, community-based health clinics, and athletic organizations in underserved communities to focus upon the specific and direct needs of underserved youth.

Pediatric mTBI researchers could consider some of the following specific examples for how to address SDoH and health equity in future studies. Focus groups could be conducted to identify potential SDoH needs and barriers to mTBI care. Researchers could directly evaluate the association between SDoH and recovery time from mTBI. Researchers could consider the feasibility of delivering interventions designed to target SDoH. For instance, the readability and reading ease scores of mTBI patient discharge instructions and patient understanding of the instructions might be examined. Studies could examine mTBI treatment and rehabilitation uptake, compliance, and barriers to accessing specialty care. Research designs aimed at informing the nature and scope of interventions designed to improve SDoH, health inequities, and clinical outcomes will be important. Additionally, research focused on uptake and utilization of the Guideline by healthcare providers in regards to SDoH and health equity factors will be important to examine.

Concussion researchers are encouraged to consistently and routinely measure and report complete demographic information about their samples. It seems particularly important for researchers to characterize gender, racial, and ethnic identities of their participants, as well as measure and report additional sociocultural characteristics such as SES and language proficiency. The website for the EQUATOR (Enhancing the QUAlity and Transparency Of health Research) network hosts additional templates for reporting guidelines.^87^ *Annals of Behavioral Medicine* recently published instructions for authors reporting on study sample socioeconomic and sociodemographic characteristics that may serve as a guide for mTBI research.^88^ Further, researchers are encouraged to measure and describe their samples in terms of relevant SDoH such as health literacy surrounding mTBI, insurance status, access, and barriers to primary care for mTBIs, as well as access and barriers to specialty concussion care.

As of about 2000, SDoH were rarely screened for in pediatric primary care settings.^89^ Encouragingly, there has been increased emphasis and considerable research interest related to screening for SDoH and related risk factors in the context of primary care,^90^ community health,^91^ and pediatric emergency medicine^92^ settings. Still, limitations in the available screening tools and resources have been highlighted along with the need for continued development and refinement of effective and practical tools.^93^ The American Academy of Pediatrics recommends screening during all patient encounters and provides a website with screening resources.^94^ It is unknown how frequently, if at all, SDoH are assessed in specialty concussion clinics—and this appears to be an important area of future research. Researchers can examine SDoH and attrition from treatment trials and address disparities accordingly.

Funding agencies and organizations are encouraged to emphasize SDoH in concussion and brain injury research. Funders could consider encouraging researchers to collect and report SDoH in their research studies. For example, the PhenX measures for social determinants of health (SDoH) project, supported by the National Institute on Minority Health and Health Disparities, developed a collection of common data elements to improve the quality and consistency of SDoH data collection.^95^ Additionally, funders could consider developing targeted mechanisms or opportunities regarding the potential role of and means to address SDoH and health inequities for pediatric mTBI diagnosis and clinical care.

## Conclusions

Over the past two decades, there has been extraordinary advancement in knowledge about pediatric mTBI. This has included, for example, many studies published examining potential risk or vulnerability factors associated with worse outcome or slower recovery from pediatric mTBI,^96–101^ as well as substantial efforts to develop and validate evidence-supported treatment and rehabilitation approaches for pediatric mTBI, such as exercise-based rehabilitation.^102-104^ The development and publication of the Guideline was a synthesis of this rapidly evolving clinical and scientific knowledge, with the dissemination and implementation goal of improving the treatment and management of pediatric mTBI. With that said, SDoH and health equity have been significantly underrepresented in the clinical research literature, including the literature underlying the Guideline.

A major priority for the field is to better understand the role and relevance of SDoH and health inequities for pediatric mTBI diagnosis and clinical management. Moreover, it is a priority to determine the means and methods that can be used to address SDoH and health inequities and to implement those means and methods, with the goal of promoting health equity. Results of this content analysis revealed that SDoH are underrepresented as important, meaningful, or primary variables of interest directly addressed in the studies that formed the basis for the prevailing Guideline for managing pediatric mTBI and the recommendations for diagnosis, prognosis, and treatment contained therein. SDoH have not represented a primary framework by which researchers designed studies or interpreted results. SDoH are relevant and important for the diagnosis and management of pediatric mTBI. We need more work in the area to inform future updated guidelines.

## Transparency, Rigor, and Reproducibility Summary

This review was not pre-registered. This review was designed as a secondary, content analysis of clinical studies that were identified in a large systematic review^34^ that was conducted as part of the Centers for Disease Control and Prevention's effort to develop the first clinical management guideline in the United States for pediatric mTBI, which was published as the “Centers for Disease Control and Prevention Guideline on the Diagnosis and Management of Mild Traumatic Brain Injury Among Children.”^33^ The coding sheet for this content analysis was developed by the authors and is provided in the supplemental materials that accompany this manuscript. Two co-authors independently coded every article and discrepancies were resolved by discussion. The results of this content analysis (i.e., the study coding results) are presented in the Tables 1 and 2 and Supplementary Tables S1 and S2. The specific quotes from each manuscript that were coded are also identified and provided in the supplementary materials (i.e., actual quote is reproduced, and the section and pages from the source article are identified).

## Supplementary Material

Supplemental data

Supplemental data
