# Supplementary material for: Social Determinants of Health and Health Equity in the Diagnosis and Management of Pediatric Mild Traumatic Brain Injury: A Content Analysis of Research Underlying Clinical Guidelines
Source: J Neurotrauma. 2023 Sep 29;40(19-20):1977–89. doi: 10.1089/neu.2023.0021 (PMC10541940; doi:10.1089/neu.2023.0021)
Supplement: Supplemental data [file Supp_TableS1.docx]

**Supplementary Table 1. Study Coding Sheet**

| **First Author:** | **PMID:** |
| --- | --- |
| **CDC Guideline Clinical Question:** | **Coder:** |

| **Social Determinants of Health**  **Domains And Subcategories** | **Did study address?** | | | |
| --- | --- | --- | --- | --- |
| **Economic Stability** | | | | |
| Employment security / stable employment | Yes | | | No |
| Food insecurity | Yes | | | No |
| Ability to afford healthy food | Yes | | | No |
| Ability to afford health care | Yes | | | No |
| Ability to afford childcare | Yes | | | No |
| Housing instability | Yes | | | No |
| People with disabilities/injuries and their ability to work | Yes | | | No |
| Poverty | Yes | | | No |
| Employment programs / career counseling | Yes | | | No |
| **Education Access and Quality** | | | | |
| Early childhood education and development | Yes | | | No |
| Enrollment in higher education | Yes | | | No |
| High school graduation | Yes | | | No |
| Language and literacy | Yes | | | No |
| Whether children are from low-income families | Yes | | | No |
| Whether children have disabilities | Yes | | | No |
| Whether children experience social discrimination (i.e., bullying) | Yes | | | No |
| Whether children live in places with poorly performing schools | Yes | | | No |
| Whether families can afford to send their children to college | Yes | | | No |
| **Health Care Access and Quality** | | | | |
| Access to health care | Yes | No | | |
| Access to primary care | Yes | No | | |
| Health literacy | Yes | No | | |
| Health and/or dental insurance | Yes | No | | |
| Ability to afford health care services and medication | Yes | No | | |
| Access to health care service recommendations/referrals (i.e., cancer screenings) | Yes | No | | |
| Access to preventative care | Yes | No | | |
| Access / transportation to healthcare providers | Yes | No | | |
| **Neighborhood and Built Environment** | | | | |
| Access to foods that support healthy eating patterns | Yes | | No | |
| Crime and violence | Yes | | No | |
| Environmental conditions (unsafe air or water) | Yes | | No | |
| Quality of housing | Yes | | No | |
| Racial residential segregation | Yes | | No | |
| Other health and safety risks in neighborhood | Yes | | No | |
| Workplace health and safety risks (i.e., second hand smoke, loud noises) | Yes | | No | |
| Biking and walking accessibility (sidewalks and bike paths) | Yes | | No | |

| **Social and Community Context** | | |
| --- | --- | --- |
| Civic participation | Yes | No |
| Discrimination / Racism | Yes | No |
| Parental incarceration | Yes | No |
| Social cohesion | Yes | No |
| Unsafe neighborhoods | Yes | No |
| Trouble affording needs | Yes | No |
| Societal attitudes and norms (i.e., racism, distrust of government) | Yes | No |
| Depression or anxiety in family caregivers | Yes | No |
| Positive versus negative relationships at home | Yes | No |
| Positive versus negative relationships at work | Yes | No |
| Positive versus negative relationships in community | Yes | No |

**Sample Description**

| Sample size (N): |  |
| --- | --- |
| Mean age, SD: |  |
| Age range: |  |
| Gender composition: |  |
| Racial composition: |  |
| Ethnic composition: |  |

**Summary Variables**

| *Health Equity Variable* | *No mention* | *Demographic category only* | *Examined in depth* |
| --- | --- | --- | --- |
| Race | □ | □ | □ |
| Ethnicity | □ | □ | □ |
| Culture | □ | □ | □ |
| Socioeconomic Status | □ | □ | □ |
| Language | □ | □ | □ |

**Comments/Notes**

Please include any comments or notes from the article. Please elaborate on any ‘yes’ responses, include the page numbers from the study, and consider copying the actual text excerpt.
