# Supplementary material for: Social Determinants of Health and Health Equity in the Diagnosis and Management of Pediatric Mild Traumatic Brain Injury: A Content Analysis of Research Underlying Clinical Guidelines
Source: J Neurotrauma. 2023 Sep 29;40(19-20):1977–89. doi: 10.1089/neu.2023.0021 (PMC10541940; doi:10.1089/neu.2023.0021)
Supplement: Supplemental data [file Supp_TableS2.docx]

**Supplementary Table 2. Quotes from the Articles Relating to Social Determinants of Health (organized alphabetically by first author for each Social Determinants of Health [SDoH] Domain)**

| **First**  **Author** | **Year** | **PMID** | **SDoH Domain** | **SDoH Subcategory** | | **Page**  **Number** | **Article**  **Section** |
| --- | --- | --- | --- | --- | --- | --- | --- |
| Blume | 2012 | 22144708 | Economic Stability | Ability to afford health care | | 33 | Results |
|  | Quote: “Compared with patients with mTBI or AI, patients with moderate/ severe TBI were more likely to be non-white, ***lack private health insurance***, ***have lower income households***, and have less educated parents, reflecting the population at the Philadelphia site.”  Table 1: Provides insurance status [None, Medicaid, Private, or Other] | | | | | | |
| Hessen | 2008 | 18183508 | Economic Stability | People with disabilities/injuries and their ability to work | | 41 | Methods |
|  | Quote: “Data were also collected regarding possible confounding factors including pre-and post-injury somatic and mental health and highest level of education and ***current work status***.”  Quote: “Three patients (7.3%) reported concentration problems at school ***and three (7.3%) had problems with reading or writing prior to the head injury***. In addition, ***two of the patients (4.9%) had learning problems at school pre-injury***. None of the patients were diagnosed with a pre-injury diagnosis of ADHD or Conduct disorder.”  Table 1: Lists 20% of the participants had a ***psychological or somatic illness before the trauma***, 24% of the participants had a ***period of illness with absence from school within 2 years before injury***, and 12% ***received disability benefit due to somatic or psychological illness***. | | | | | | |
| Levin | 2008 | 18518697 | Economic Stability | Poverty | | 462 | Introduction |
|  | Quote: “Based on evidence that the family environment and extra-cranial injury can influence the effects of moderate to severe TBI on neuropsychological outcome in children, we explored whether ***SES***, the severity of injury to body regions other than the head, a preinjury psychiatric disorder, or patient age and sex influenced recovery in those with an mTBI.”  Quote: “***The Hollingshead four-factor index, which measures SES***, was calculated based on the mother and father’s years of education and occupation.” | | | | | | |
| Max | 2013 | 24247854 | Economic Stability | Employment security/ stable employment | | 277 | Results |
|  | Quote: “As hypothesized, ***SES***, ***psychosocial adversity***, and estimated pre-injury academic functioning showed significance.”  Quote: “The Four-Factor Index was used to assess ***socioeconomic status (SES).*** Classification is based on scores derived from a formula involving both the paternal and maternal educational and occupational levels. Higher scores indicate higher SES, ranging from 8 to 66.”  Quote: “We modeled our psychosocial adversity index after that used in an important early study of pediatric TBI. The assessment involved six areas, and, for each area that suggested adversity, a score of 1 was given; for each area where there was no adversity, a score of 0 was given. The areas are 1) child not living with biological or adoptive parents; 2) sibship of at least 4 children or a person:room ratio exceeding 1; 3) admission of the child into the care of local authorities because of family difficulties; 4) maternal “malaise inventory” score of ≥7; 5) paternal criminality; and 6) ***father or mother with an unskilled or semi-skilled job***.” | | | | | | |
| Max | 2013 | 24026712 | Economic Stability | Employment security/ stable employment | | 193 | Results |
|  | Quote: “None of the other demographic or psychosocial variables, including age at injury, gender, ***socioeconomic status***, race, pre-injury adaptive functioning, pre-injury family functioning, family psychiatric history, or ***pre-injury psychosocial adversity*** discriminated between groups.”  Quote: “We used a psychosocial adversity index that was very similar to that used in an important early study of pediatric TBI. Six areas were assessed… ***6) father or mother with an unskilled or semiskilled job***.” | | | | | | |
| Rivara | 2011 | 22025592 | Economic Stability | Ability to afford healthcare | | 1131 | Methods |
|  | Quote: “…we also obtained self-report data on the potential confounders of race/ethnicity, ***insurance***, ***household income***, and respondent education.” | | | | | | |
| Taylor | 2015 | 25629259 | Economic Stability | Poverty | | 304 | Methods |
|  | Quote: “***Socioeconomic status*** defined as average of sample z-scores for year of maternal education, median family income based on census data for the neighborhood of residence, and the Duncan Socioeconomic Index (a measure of occupational status).” | | | | | | |
| Zonfrillo | 2014 | 24294826 | Economic Stability | | Ability to afford healthcare | 723 | Results |
|  | Quote: “Poor functioning was significantly associated with… ***Medicaid insurance versus private insurance*** (RR [95% CI] =2.22[1.21,4.06]) at 12 months…”  Quote: “Poor functioning was significantly associated with… ***low annual household income of <$30,000 versus >$100,000*** (RR [95% CI] =2.73[1.28,5.83], 3.10[1.40,6.86]) at 3 and 12 months respectively…” | | | | | | |
| Agrawal | 2005 | 16156226 | Education Access | | Early childhood education and development | 170 | Discussion |
|  | Quote: “Wrightson, et al., prospectively studied the effect of minor head injury on preschool children by using neuropsychological tests and found that injury in the preschool years seemed to affect the process of learning to read. The authors stressed that if ***early identification and diagnosing these children could be made, teachers could become aware of this and remedial teaching could be made available*** to them.” | | | | | | |
| Babikian | 2011 | 21813031 | Education Access | | Whether children have disabilities | 887 | Introduction |
|  | Quote: “This is particularly problematic given that accidental injury in general, and noninflicted TBI in particular, is reportedly associated with several pre-existing risk factors, including ***psychiatric disorders such as attention deficit/hyperactivity disorder (AD/HD)***. It is possible that the adverse effects of mild TBI on neurocognition found in some studies may reflect the effect of pre-injury conditions such as AD/HD and learning disabilities that are associated with poor neurocognitive function.”  Quote: “Initial data were collected shortly after injury (at the 1 month post-injury visit) to ensure that pre-injury information, including ***history of learning, school, and behavior problems (e.g., attention and/or conduct problems*)**, use of alcohol, and prior injury would be available and least biased” | | | | | | |
| Babikian | 2013 | 23157821 | Education Access | | Whether children have disabilities | 150 | Results |
|  | Quote: “Of the demographic/premorbid variables, ***academic achievement, presence of a diagnosed learning problem***, and parental education all predicted neurocognitive impairment in univariate models.”  Quote: “In this multivariate model, which included ***premorbid academic functioning and*** ***learning problems diagnoses***, *post-injury behavioral/ emotional problems, and neurocognitive performance* at 1-month post-injury, only the latter variable maintained independent statistical significance as a predictor, with the overall model explaining 55% of the variance” | | | | | | |
| Fay | 2010 | 19835663 | Education Access | | Whether children have disabilities | 96 | Methods |
|  | Quote: “Children were not excluded for ***premorbid learning difficulties or attention problems***.” | | | | | | |
| Grubenhoff | 2010 | 20819901 | Education Access | | Language and literacy | 689 | Methods |
|  | Quote: “***English-speaking children of Spanish-speaking parents were included***; consent was obtained from the parents in Spanish.”  Quote: “We adapted the questions of the graded symptom checklist so that ***the language was age-appropriate***.” | | | | | | |
| Hessen | 2008 | 18183508 | Education Access | | High school graduation | 40 | Results |
|  | Quote: “Average length of education was 13.5 years (SD=2.9), ***which is slightly above the normative educational level of 13 years*** for 30-39 year old Norwegians.” | | | | | | |
| O’Connor | 2012 | 22729979 | Education Access | | Whether children have disabilities | 267 | Methods |
|  | Quote: “Additionally, parents reported on the child’s ***preinjury history of learning disability and emotional functioning, which included questions regarding history of anxiety, attention-deficit/hyperactivity, behavioral concerns, and any additional emotional concerns***.” | | | | | | |
| Ponsford | 1999 | 10407209 | Education Access | | Whether children have disabilities | 362 | Methods |
|  | Quote: “Parents and children had to speak English to complete the study procedures. Children with a history of previous head injury or ***other psychological or neurological problems and/or learning difficulties, as determined from parental interview, were not excluded because these factors have been shown to be associated with poorer outcomes*** in some previous studies of mild THI in children.” | | | | | | |
| Ponsford | 2001 | 11731651 | Education Access | | Whether children have disabilities | 1298 | Methods |
|  | Quote: “Children with a history of previous head injury or ***other psychological or neurologic problems or learning difficulties were not excluded because these factors have been shown to be associated with poorer outcomes*** in some previous studies of mild TBI in children.” | | | | | | |
| Schatz | 2006 | 16143492 | Education Access | | Whether children have disabilities | 95 | Results |
|  | Quote: “Demographic variables were analyzed to establish between-group homogeneity, with no differences noted between concussion history groups on age, education, handedness, history of special education, or ***diagnosis of learning disability***.” | | | | | | |
| Taylor | 2015 | 25629259 | Education Access | | Whether children have disabilities | 303 | Methods |
|  | Quote: “To increase generalizability, ***children with histories of learning disabilities***, attention-deficit/hyperactive disorder, or other behavior disorders were not excluded from either group.” | | | | | | |
| Babikian | 2011 | 21813031 | Health Care Access | | Access/ transportation to healthcare providers | 888 | Methods |
|  | Quote: “Patients were studied prospectively and assessments were ***conducted in the homes of the children*** to further avoid methodological and sampling biases typically present in studies of retrospective or clinical samples” | | | | | | |
| Blume | 2012 | 22144708 | Health Care Access | | Health and/or dental insurance | 33 | Results |
|  | Quote: “Compared with patients with mTBI or AI, patients with moderate/ severe TBI were more likely to be non-white, ***lack private health insurance***, have lower income households, and have less educated parents, reflecting the population at the Philadelphia site.”  Table 1: Provides type of insurance [None, Medicaid, Private, or Other] | | | | | | |
| Gagnon | 2004 | 15597030 | Health Care Access | | Access/ transportation to healthcare providers | 396 | Methods |
|  | Quote: “***All evaluations took place in the children’s homes to minimize traveling inconvenience*** and school absenteeism.” | | | | | | |
| Rivara | 2011 | 22025592 | Health Care Access | | Health and/or dental insurance | 1131 | Methods |
|  | Quote: “…we also obtained self-report data on the potential confounders of race/ethnicity, ***insurance***, household income, and respondent education.”  Table 1: Provides health insurance status [None, Medicaid, Private, Tricare/CHAMPUS, Basic health, or Unknown] | | | | | | |
| Zonfrillo | 2014 | 24294826 | Health Care Access | | Health and/or dental insurance | 723 | Results |
|  | Quote: “Poor functioning was significantly associated with… ***Medicaid insurance versus private insurance*** (RR [95% CI] =2.22[1.21,4.06]) at 12 months…” | | | | | | |
| Babikian | 2013 | 23157821 | Social and Community Context | | Positive versus negative relationships at home | 148 | Methods |
|  | Quotes: “Study variables used as predictors of outcome in the analyses were grouped as follows: (i) clinical (e.g., injury severity, type of injury, number of head injuries, severity of recent injury, number of concussive symptoms from recent injury, with the latter two referring only to the TBI group),(ii) premorbid/demographic (e.g., parent report of premorbid behavioral/emotional problems, premorbid academic achievement, parental education, history of diagnosed problems),and (iii) post-injury functioning (e.g., parent report of post-injury behavioral/emotional problems, ***family stress***, cognitive impairment at 1-month post-injury).” | | | | | | |
| Barlow | 2010 | 20660554 | Social and Community Context | | Depression or anxiety in family caregivers | 376 | Measures |
|  | Quote: “The Brief Symptoms Inventory (BSI) was used to ***assess maternal psychological adjustment*** at 1, 6, and 12 months after injury” | | | | | | |
| Barlow | 2010 | 20660554 | Social and Community Context | | Positive versus negative relationships at home | 376 | Measures |
|  | Quote: “The 12-item General Functioning Scale (GFS) of the McMaster Family Assessment Device (FAD) was used as a summary measure of ***family functioning***.” | | | | | | |
| Max | 2013 | 24247854 | Social and Community | | Parental incarceration | 276 | Methods |
|  | Quote: “The assessment involved six areas, and, for each area that suggested adversity, a score of 1 was given; for each area where there was no adversity, a score of 0 was given. The areas are…***5) paternal criminality***…” | | | | | | |
| Max | 2013 | 24247854 | Social and Community | | Depression or anxiety in family caregivers | 274 | Methods |
|  | Quote: “Table 1 presents data on demographics (age, gender, SES), pre-injury psychosocial variables (pre-injury lifetime psychiatric status, adaptive functioning, family functioning, ***family psychiatric history***, psychosocial adversity), and injury indices (GCS scores, depressed skull fracture incidence, mechanism of injury).” | | | | | | |
| Max | 2013 | 24247854 | Social and Community | | Positive versus negative relationships at home | 274 | Methods |
|  | Quote: “Table 1 presents data on demographics (age, gender, SES), pre-injury psychosocial variables (pre-injury lifetime psychiatric status, adaptive functioning, ***family functioning***, family psychiatric history, psychosocial adversity), and injury indices (GCS scores, depressed skull fracture incidence, mechanism of injury).”  Quote: “The assessment involved six areas, and, for each area that suggested adversity, a score of 1 was given; for each area where there was no adversity, a score of 0 was given. The areas are 1) child not living with biological or adoptive parents; 2) sibship of at least 4 children or a person:room ratio exceeding 1; ***3) admission of the child into the care of local authorities because of family difficulties***; 4) maternal “malaise inventory” score of >7; 5) paternal criminality; and 6) father or mother with an unskilled or semi-skilled job.” | | | | | | |
| Max | 2013 | 24026712 | Social and Community Context | | Parental incarceration | 190 | Methods |
|  | Quote: “We used a psychosocial adversity index that was very similar to that used in an important early study of pediatric TBI. Six areas were assessed… ***5) paternal criminality***.” | | | | | | |
| Max | 2013 | 24026712 | Social and Community Context | | Depression or anxiety in family caregivers | 189 | Methods |
|  | Quote: “Demographic details (age, gender, socio-economic status), pre-injury psychosocial variables (pre-injury life-time psychiatric status, adaptive functioning, family functioning, ***family psychiatric history ratings***, psycho-social adversity), and injury indices (GCS scores, depressed skull fracture incidence, mechanism of injury) are provided in Table 1.  Quote: “We used a psychosocial adversity index that was very similar to that used in an important early study of pediatric TBI. Six areas were assessed… ***4) maternal “malaise inventory” score of >7***…” | | | | | | |
| Max | 2013 | 24026712 | Social and Community Context | | Positive versus negative relationships at home | 189 | Methods |
|  | Quote: “Demographic details (age, gender, socio-economic status), pre-injury psychosocial variables (pre-injury life-time psychiatric status, adaptive functioning, ***family functioning***, family psychiatric history ratings, psycho-social adversity), and injury indices (GCS scores, depressed skull fracture incidence, mechanism of injury) are provided in Table 1.  Quote: “We used a psychosocial adversity index that was very similar to that used in an important early study of pediatric TBI. Six areas were assessed… 1) child not living with biological or adoptive parents; 2) sibship of at least 4 children, or a Person: Room ratio exceeding 1; ***3) admission of the child into the care of the local authority because of family difficulties;*** 4) maternal “malaise inventory” score of >7; 5) paternal criminality…” | | | | | | |
| O’Connor | 2012 | 22729979 | Social and Community Context | | Depression or anxiety in family caregivers | 267 | Methods |
|  | Quote: “Baseline family functioning was measured with the General Functioning Scale of the McMaster Family Assessment Device (FAD; Miller, Epstein, & Bishop, 1985), which provides information on overall health and ***psychopathology within the family***…” | | | | | | |
| O’Connor | 2012 | 22729979 | Social and Community Context | | Positive versus negative relationships at home | 267 | Methods |
|  | Quote: “Baseline ***family functioning*** was measured with the General Functioning Scale of the McMaster Family Assessment Device (FAD; Miller, Epstein, & Bishop, 1985), which provides information on overall health and psychopathology within the family…” | | | | | | |
| Ponsford | 1999 | 10407209 | Social and Community Context | | Positive versus negative relationships at home | 369 | Results |
|  | Quote: “The input variables were gender, history of previous head injury, previous learning difficulties, previous neurological/psychiatric problems, ***premorbid stressors***, and PTA duration.” | | | | | | |
| Ponsford | 2001 | 11731651 | Social and Community Context | | Positive versus negative relationships at home | 1299 | Results |
|  | Quote: “However, there was a significantly higher incidence of previous head injury in this problem subgroup and a ***higher incidence of premorbid stressors, such as family breakdown***, preexisting psychiatric or neurologic problems, and learning difficulties.” | | | | | | |
| Rivara | 2011 | 22025592 | Social and Community Context | | Positive versus negative relationships at home | 1131 | Measures |
|  | Quote: “***We used the self-report McMaster Family Assessment Device to measure family functioning and emotional relationships within the family***.” | | | | | | |
| Smyth | 2014 | 23992222 | Social and Community Context | | Positive versus negative relationships at home | 74 | Methods |
|  | Quote: “The Stressful Life Events Questionnaire (SLEQ)… contained 10 questions about ***familial and personal stressors experienced in the child’s lifetime…*** including previous hospitalizations, life threatening illness, major illness, major accidents, episodes of physical force (physical abuse, physical assault by adult/sibling/peer resulting in significant injury (e.g. stitches, broken bone, severe bruising), deaths (pet/friend/second-degree relative or first degree relative), life threatening illnesses of someone close, breakups in family, moving homes, and changing schools in the middle of school year.” | | | | | | |
